# Supplementary material for: MetaRibo-Seq measures translation in microbiomes
Source: Nat Commun. 2020 Jun 29;11:3268. doi: 10.1038/s41467-020-17081-z (PMC7324362; doi:10.1038/s41467-020-17081-z)
Supplement: Supplementary file 10 — Supplementary Data 7 [file 41467_2020_17081_MOESM10_ESM.zip › File2/Confidence_High_Taxonomy/426886_out.krona.html]

Javascript must be enabled to view this page.

members
magnitude
magnitudeUnassigned
count
unassigned
taxon
rank

426886\_out

1

2759
1
superkingdom

kingdom
1
4751

451864
1
subkingdom

phylum
4890
1

147538
1
subphylum

class
1
147550

222544
1
subclass

1
5151
order

family
1
5152

genus
1
29907

150359
1

SRS017307\_contig\_number\_contig-100\_2450.135202
species
